# Exploring Lemon Industry By-Products for Polyhydroxyalkanoate Production: Comparative Performances of *Haloferax mediterranei* PHBV vs. Commercial PHBV

**DOI:** 10.3390/polym18030340

**Published:** 2026-01-27

**Authors:** Salvador García-Chumillas, María Nicolás-Liza, Fuensanta Monzó, Pablo-Manuel Martínez-Rubio, Alejandro Arribas, Rosa María Martínez-Espinosa, Ramón Pamies

**Affiliations:** 1 Cetec Biotechnology S. L., Avda Europa, 4, 30840 Alhama de Murcia, Spain; m.nicolas@ctcalzado.org; 2 Technological Centre of Footwear and Plastic of the Region of Murcia (CETEC) Avda Europa, 4, 30840 Alhama de Murcia, Spain; f.monzo@ctcalzado.org (F.M.); a.arribas@ctcalzado.org (A.A.); 3 Grupo de Ciencia de Materiales e Ingeniería Metalúrgica, Universidad Politécnica de Cartagena, Dr. Flemming s/n, 30202 Cartagena, Spain; pablo.mrubio@upct.es (P.-M.M.-R.); ramon.pamies@upct.es (R.P.); 4 Biochemistry and Molecular Biology and Edaphology and Agricultural Chemistry Department, Faculty of Sciences, University of Alicante, 03007 Alicante, Spain; rosa.martinez@ua.es

**Keywords:** poly(3-hydroxybutyrate-co-3-hydroxyvalerate), haloarchaea, residues valorisation, circular economy, bioplastics, biodegradable plastics

## Abstract

This study investigates the valorisation of lemon industry by-products as carbon sources to produce poly(3-hydroxybutyrate-co-3-hydroxyvalerate) (PHBV) using the halophilic archaeon *Haloferax mediterranei*. The resulting polymer (HFX PHBV) was supplemented with nucleating agents (orotic acid, boron nitride, and theobromine) and compared with a commercial PHBV grade (Enmat Y1000) under identical conditions. Fermentation strategies were optimised by varying the lemon by-product concentration, inoculum size, and nutrient stoichiometry (C:N:P ratios), followed by scaleup in a 2 L bioreactor. A 11% (*v*/*v*) lemon by-product combined with a 5% (*v*/*v*) inoculum yielded the highest productivity under minimal medium conditions (2.127 g/L PHBV), while enriched media further enhanced the polymer accumulation (up to 3.250 g/L PHBV). A comparative characterisation of HFX PHBV and Enmat Y1000, using NMR, TGA, MFR, DSC, Raman spectroscopy, XRD, and DMA, revealed that HFX PHBV exhibited lower crystallinity, increased flexibility, and a high hydroxyvalerate content (27.4%), which conferred improved ductility. Investigation of nucleating agents demonstrated that orotic acid was the most effective at enhancing the crystallisation kinetics. Overall, this study demonstrates an efficient PHBV production process based on waste valorisation, yielding a biopolymer with competitive physicochemical properties relative to a commercial standard, and provides integrated solutions to the global challenges of plastic pollution and food waste.

## 1. Introduction

The world is currently facing numerous challenges to ensure its long-term sustainability. Food waste is intricately related to ongoing population growth and consumption habits, and it is becoming a critical global issue [1]. This problem is linked to plastic pollution, as plastic is the most common material for food packaging [2]. Plastic contamination has spread to all ecosystems, from marine (including oceans, seas, and even Antarctica) to terrestrial [3,4,5,6,7,8,9], and it has even been found in animal tissues [10,11]. A holistic approach to these two interconnected problems would be to use agrifood waste as a feedstock to produce biobased and biodegradable plastics with applications in food packaging [12,13].

One of the most well-studied bioplastics for this purpose is the polyhydroxyalkanoate (PHA) group. PHAs are biodegradable and biobased aliphatic polyesters. In this group, poly(3-hydroxybutyrate-co-3-hydroxyvalerate) (PHBV) is one of the most studied polymers. Commercial PHBV is used more because of its better malleability and flexibility compared to poly(3-hydroxybutyrate) (PHB) [14]. When the concentration of the copolymer 3-hydroxyvalerate (3HV) is at least 20%, PHBV has comparable properties to those of polypropylene [15,16]. Some of these properties are physicochemical and mechanical features, as well as thermoplasticity [12].

PHAs are produced intracellularly and accumulated as granules as part of a microbial strategy to store energy and carbon [12,17,18,19]. The synthesis of PHAs occurs when the microorganisms suffer a nutrient-limiting concentration of nitrogen, phosphorus, sulphur, or oxygen and excess carbon sources [18,20,21,22,23]. This mechanism is used by a huge number of different microorganisms, including bacteria [24,25], yeast [26,27], algae [28,29], and archaea [21,30]. However, PHBV is produced by a lower number of microorganisms, and if 3HV precursors are not used during the fermentation, this number decreases. The most promising microorganism able to synthesise PHBV without a 3HV precursor addition is *Haloferax mediterranei* [20,31] due to its versatile metabolism, its ability to use a wide range of single-carbon sources and its industrial waste, its high growth rate compared to other microorganisms, and its genetic stability. Moreover, considering that *H. mediterranei* is a haloarchaeon, sterile conditions can be less stringent during fermentation processes due to the high salt concentration of the culture media used [29,32,33].

The slow crystallisation rate of PHAs is a major challenge for high-throughput applications, as it can lead to suboptimal crystalline structures and reduced mechanical properties [34]. To overcome this concern, the plastics industry has long used nucleating agents to enhance the crystallisation kinetics in thermoplastics. Therefore, finding more sustainable nucleators for PHBV is crucial for their commercialisation.

For PHAs, several effective nucleating agents have been found, with cyclic imide-containing molecules being particularly common. Orotic acid (OA), cyanuric acid (CA), uracil, and thymine are all known to nucleate PHAs, and both OA and CA have shown high efficiency for PHBHHx copolymers [35]. OA was found to nucleate PHBHHx copolymers three times faster than boron nitride, a common inorganic nucleator [36]. This striking efficiency has prompted this investigation into whether OA performs equally well in high-hydroxyvalerate PHBV. With this work, we contribute to the optimisation of the production of PHBV by using microbial cells and valorising agrifood by-products, thereby contributing to global sustainability.

## 2. Materials and Methods

### 2.1. Microorganism and Inoculum Preparation

The haloarchaeon *Haloferax mediterranei* R4 was used as a PHBV producer microorganism for all the experiments. This strain is part of the microbial collection in the hands of the Applied Biochemistry Research group (University of Alicante, Spain). The microbial cells were maintained on the *Halobacterium* medium prescribed by the ATCC, ATCC 1176, with the following composition (g/L): 156.0 NaCl; 13.0 MgCl_2_·6H_2_O; 20.0 MgSO_4_·7H_2_O; 1.0 CaCl_2_·2H_2_O; 4.0 KCl; 0.2 NaHCO_3_; and 0.5 NaBr, as marine salts, and 0.5 KH_2_PO_4_; 0.005 FeCl_3_; 5.0 yeast extract; and 1.0 glucose. Working stocks prepared from colony isolation were preserved at −80 °C in cryovials. To initiate the seed culture, cells were reactivated in 50 mL of ATCC 1176 medium and incubated for 24 h at 37 °C with shaking at 170 rpm. The seed culture was then transferred to the inoculum flasks of 500 mL containing 100 mL of the mentioned culture medium for 24 h at 37 °C with shaking at 170 rpm.

### 2.2. Determination of Cell Growth

Cell growth was analysed spectrophotometrically during the experiments by measuring the optical density at 600 nm (OD_600_) using the LLG-uniSPEC 4 spectrophotometer (LLG, Meckenheim, Germany).

### 2.3. Agrifood Residues as a Carbon Source for PHBV Production

Lemon by-products are sugar-rich streams from different processes from the industry and supplied by the “Centro Tecnológico Nacional de la Conserva y Alimentación (CTNC)”. The aim was to transform these agro-industrial by-products into fermentable sugars for the biosynthesis of PHBV, contributing to a circular bioeconomy. Characterization of lemon by-products in terms of their sugar composition was provided by the CTNC (Table 1), revealing the presence of significant concentrations of fermentable carbohydrates.

These characterisation results confirmed the feasibility of using lemon by-products as a carbon-rich feedstock for microbial growth and PHBV accumulation. Building on these findings, two screening approaches and a comparison between minimal and enriched media were undertaken to further evaluate the biotechnological potential of this substrate, with lemon by-products as the sole carbon source.

#### 2.3.1. First Screening (Lemon By-Product Concentration)

In the first screening, different concentrations of lemon by-product were tested. In this experimentation, to assess the microbial growth kinetics and PHBV production under controlled nutrient conditions, only a minimal culture medium was employed. The components of this minimal medium are 4 g/L KNO_3_ as the nitrogen source; 0.5 g/L NaH_2_PO_4_·2H_2_O as the phosphorous source; 156.0 g/L NaCl; 27.67 g/L MgCl_2_·6H_2_O; 19.30 g/L MgSO_4_·7H_2_O; 0.97 g/L CaCl_2_·2H_2_O; 4.0 g/L KCl; 0.13 g/L NaHCO_3_; 0.47 g/L NaBr; and 5 mg/L FeCl_3_. The lemon by-product was the carbon source. All experiments were conducted using 200 mL of cell culture in 1000 mL flasks at 42 °C and pH 7.2. The cultivation time finished once the cultures reached the first stage of the stationary phase in a horizontal incubator (BioSan, Riga, Latvia) with a shaking platform (170 rpm). Also, the inoculum OD_600_ was 4.16 in the stationary phase, and its rate was 5% (*v*/*v*).

In the first screening, to evaluate the effect of carbon source on microbial growth and PHBV production, a series of culture media (ML1–ML5) with progressively increasing proportions of lemon by-products (4.5, 11, 14, 17.5 and 20% *v*/*v*) was prepared.

#### 2.3.2. Second Screening (Inoculum Percentage Scanning)

After the initial screening, the three conditions that showed the most promise were selected for a second screening phase, using the minimal culture medium. This new set of experiments was designed to evaluate the influence of the inoculum size on microbial growth and polymer biosynthesis.

The conditions (ML2–ML11) maintained constant carbohydrate profiles derived from lemon by-products from the first screening (11, 14, and 17.5% *v*/*v*) while varying the inoculum percentage: 2.5, 5.0, and 10.0% (*v*/*v*). The combination of these two parameters is detailed in Table 2. By keeping the lemon by-product concentration fixed within each triplet, the specific impact of the inoculum percentage can be isolated. All experiments were carried out at 42 °C at pH 7.2 for 120 h, using 200 mL of cell culture in 1000 mL flasks, with shaking at 170 rpm and in triplicate.

#### 2.3.3. Carbon–Nitrogen–Phosphorus (C:N:P) Ratio Experiments

In the carbon–nitrogen–phosphorus (C:N:P) ratio research, the influence of the nutrient availability on the microbial performance and PHBV biosynthesis was investigated. This approach consisted of varying the C:N:P ratios to modulate the microbial growth dynamics and biopolymer production, using lemon by-products as the sole carbon source and adjusting the nitrogen and phosphorus concentrations in both minimal and enriched culture media.

Six experimental conditions were designed, as described in Table 3, maintaining the same concentration of lemon by-products (either 11% or 14% *v*/*v*) to ensure consistency in the carbon input. All conditions were inoculated with an inoculum at a final concentration of 5% (*v*/*v*). In the minimal medium, two concentrations of the phosphorus source were tested (0.1 and 0.5 g/L), while in the enriched medium, the concentration was kept constant at 0.1 g/L. By exploring a range of C:N (5.68–9.12) and C:P (40.01–254.59) ratios, this experimental setup enabled the identification of conditions that favour PHBV synthesis over cellular growth. All experiments were carried out at 42 °C and pH 7.2 for 120 h, using 200 mL of culture in 1000 mL flasks with shaking at 170 rpm, and were performed in triplicate.

### 2.4. Scaling Up the Fermentation

In the scaleup experiments, the feasibility of transferring the PHBV production process to a larger bioreactor system was evaluated, with particular focus on the kinetics of microbial growth and the point at which maximum PHBV accumulation was achieved. This stage aimed not only to confirm the scalability of the fermentation strategy using lemon by-products but also to determine the optimal fermentation time for maximising polymer productivity under controlled bioreactor conditions.

To upscale the production of PHBV using lemon by-products as raw material, a 2 L benchtop bioreactor (SOLARIS ONE 2.0, Porto Mantovano, Italy) was operated at a working volume of 1.4 L. The system was equipped with a Visiferm DO Arc 325 H0 probe for dissolved oxygen measurement and an Easyferm Bio HB Arc 325 probe for pH monitoring (both from Hamilton, Bonaduz, Switzerland). The temperature was maintained at 42 °C, and the pH was controlled at 7.2 through the automatic addition of 1 M KOH. The dissolved oxygen level was set to 20% saturation, and agitation was regulated between 400 and 700 rpm to ensure sufficient aeration and mixing. Foam formation was controlled by the initial addition of 3 mL of silicone antifoam.

The fermentation was carried out using the EL3 culture medium and was stopped after 72 h, when an increase in the dissolved oxygen percentage indicated that the cells had reached the stationary phase. Samples were collected at 24 h, 48 h, and 72 h to monitor the biomass formation and PHBV production over time. The PHBV produced during this scaleup was subsequently used for further physicochemical characterisation and performance testing.

### 2.5. PHBV Extraction

The polymer accumulated inside the cells must be extracted and purified to evaluate the yield of the fermentation process. For PHBV quantification in the flask trials, after each experiment, the cells were harvested by centrifugation at 4360× *g* for 60 min. Then, the supernatant was discarded. The remaining pellet was washed with NaCl solution at 10% (*w*/*v*), centrifuged again, and resuspended with tap water. The resuspended biomass was finally dried in an oven at 70 °C. The dried biomass was subjected to solid–liquid extraction, specifically Soxhlet extraction, using 70 mL of chloroform per sample. After extraction, PHBV samples were dried in a rotary evaporator and washed to remove lipid residues using 50 mL of methanol. Washed PHBV was then dried at 40 °C, and the recovered mass was weighed and further characterised.

Meanwhile, for PHBV quantification in the scaleup, process samples (50 mL) of the cultures were taken at different times (24, 48, and 72 h). The rest of the experimentation followed the same protocol.

### 2.6. PHBV Characterisation

As part of this study, it was necessary to characterise the polymer obtained, since its chemical composition and, more specifically, the hydroxyvalerate comonomer content affected its thermal behaviour, processability, and mechanical properties. The techniques that were chosen for this purpose, and the materials used for the experimentation, are described below.

#### 2.6.1. Materials Used

The PHBV polymers compared were the polymers from several scaleup fermentations of *H. mediterranei* using lemon by-products (HFX PHBV) and the commercially available PHBV (Y1000).

The nucleating agents chosen to enhance the polymer crystallinity were boron nitride (BN) (Sigma-Aldrich, 255475-250G, Schnelldorf, Germany), orotic acid (Sigma-Aldrich, O2750-10G, Wuxi, China) (OA), and theobromine (T) (Sigma-Aldrich, T4500-25G, Buchs, Switzerland) at 1% by weight.

#### 2.6.2. Characterisation of PHBV by Nuclear Magnetic Resonance (NMR)

The chemical structure of the copolymer HFXPHBV was confirmed by measuring the chemical shift position in ^1^H NMR spectroscopy. NMR spectra were recorded at 298 K on a Bruker Advance 400 MHz instrument. Chemical shifts (δ) are reported in parts per million (ppm) and referenced to CDCl3 (1H: 7.26 ppm; 13C: 77.0 ppm). The molar content (%) of the 3-hydroxyvalerate (3HV) unit in the copolymer was calculated from the intensity ratio of methyl groups for 3HV groups, as show in Equation (1), according to the method described by Bloembergen and Holden [37]:

(1) $$3HV\;(\%)=\;\frac{I_{7}}{I_{3}+I_{7}}\;\times \;100$$

Equation (1). Calculation of 3HV percentage.

#### 2.6.3. Thermogravimetric Analysis (TGA)

TGA of the samples was conducted to study their thermal stability, using a Netzsch TGA 209 F1 Libra apparatus (Selb, Germany). The samples were loaded into 70 μL aluminium crucibles, placed on a sensitive balance inside a furnace, and heated from 25 to 600 °C at a rate of 20 °C/min in an inert nitrogen atmosphere (gas flow rate: 20 mL/min). An isothermal step was then performed at 600 °C for 30 min in an oxidising atmosphere to remove pyrolytic residues (gas flow rate of 20 mL/min). Mass losses are registered and related to the thermal decomposition of the polymer.

#### 2.6.4. Melt Flow Rate (MFR)

The MFR was determined by measuring the mass of a molten polymer passing through a standard die using a Melt Flow Indexer (or extrusion plastometer) (CFlow de ZwickRoell (Ulm, Alemania)). Samples of HFX PHBV (5 g) were tested at 140, 145, and 150 °C, whereas Y1000 samples (5 g) were tested at 180 °C. All samples were tested under a load of 2.16 kg.

### 2.7. PHBV Extrusion and Comparison with a Commercial PHBV

#### 2.7.1. PHBV Extrusion

Prior to processing, the polymer was milled in the ultra-centrifugal mill model used in this project, which is the ZM 200 from Retsch (Haan, Germany). This work used a speed of 12,000 rpm, using the 1 mm sieve for extrusion and the 0.25 mm sieve for X-ray diffraction.

The polymer is mixed with the nucleating agent through an extrusion process. The main model used is a Twinlab 10 mm corotating twin-screw extruder from Twintech Extrusion Ltd. (Stoke-on-Trent, UK). The temperature profiles of the polymers are shown in Table 4.

#### 2.7.2. Characterisation of PHBV by Differential Scanning Calorimetry (DSC)

The DSC model used was the Perkin Elmer Pyris DSC 6 (Norwalk, CA, USA). All tests were performed in an open crucible under air, using 40 μL aluminium crucibles with 13 mg samples. The programs used are detailed in Table 5. The peak temperatures of the melting (Tm) and crystallisation (Tc) processes, as well as their enthalpies, were estimated by integrating the DSC curve in the heating step (melting) and cooling step (crystallisation).

#### 2.7.3. Raman Spectroscopy

Raman spectroscopy was used to analyse the samples by identifying the presence of nucleating agents and their distribution. For this work, a WITec alpha300 access confocal Raman spectroscopy microscope (Ulm, Germany) was equipped with 10× and 100× magnification objectives. The radiation source has a wavelength of 532 nm and a maximum nominal power of 30 mW, which can be adjusted using an attenuator. The spectrophotometer used was the WITec UHTS 300 VIS-NIR. A power of 23 mW was used in this work, and spectra were acquired using acquisition times of 0.5 s with 10 iterations. Both mappings and point measurements were taken with a 100× objective, selecting a 200 × 100 μm mapping surface consisting of 20 points per line and 10 lines.

#### 2.7.4. X-Ray Diffraction (XRD)

XRD was used to study the crystallinity of the polymer, which influences its physical properties, such as its strength and stiffness. The equipment used in this work corresponded to a Bruker D8 Advance X-ray powder diffractometer (Billerica, MA, USA), which employs CuKα emission (λ = 1.54 Å, 40 kV, 30 mA) in θ-θ configuration. In this work, we used the sample ground with a 0.25 mm sieve in a 2θ range of 3 to 70° with 0.05° intervales. The XRD patterns were obtained using a method described in the literature with the aid of DIFFRAC.SUITE v.7303 software. The calculation of the crystalline areas is based on the Farrow–Preston model [38,39,40,41].

#### 2.7.5. Dynamic Mechanical Analysis (DMA)

DMA provides key information on a polymer’s traction properties. A TA Instruments Q800 dynamic-mechanical analyser (New Castle, DE, USA) was used in this study. Tests were performed on extruded filaments measuring 1.8 mm (on average) in a tensile configuration. A miniature version of a tensile test was performed up to 18 N with a loading rate of 5 N/min, sufficient for the study of the elastic zone. From this zone, the modulus of elasticity was extracted by the linear regression of the values between 0.05 and 0.25% strain. This analysis was performed at 0, 1, 2, 3, and 7 days.

## 3. Results

### 3.1. Agrifood Residues as a Carbon Source for PHBV Production

#### 3.1.1. First Screening: Effect of Lemon By-Product Concentration on Microbial Growth and PHBV Production

In the first set of experiments, a minimal culture medium was supplemented with increasing concentrations of lemon by-products (from 4.7% to 20.0% *v*/*v*) to evaluate the use of these by-products as a carbon source by the microbial cells, where microbial growth and PHBV accumulation were monitored.

**Figure 1:**
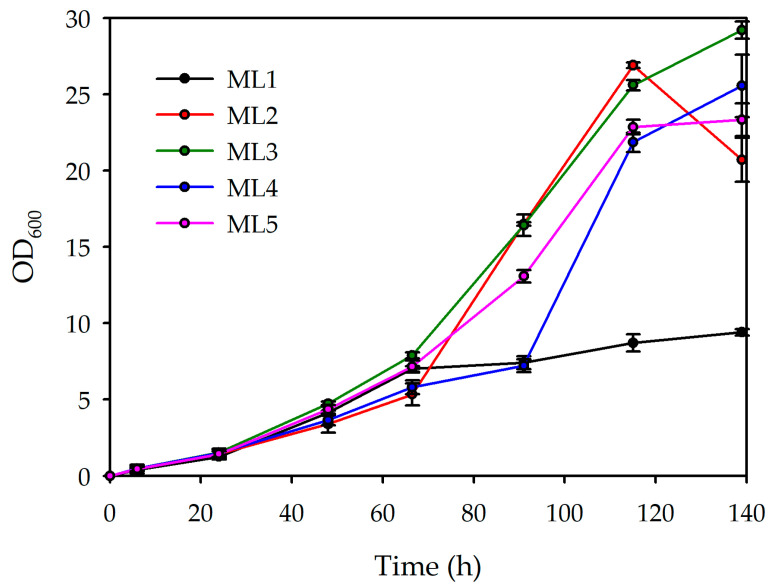
Growth curves and duplication rate obtained from each growth condition from the first screening.

The growth curves (Figure 1) of the five conditions (ML1–ML5) display distinct kinetic behaviours over the 140 h cultivation period. In the initial phase (until 40 h), all conditions exhibit similar lag and early exponential growth. Beyond this point, divergence becomes evident. ML3 and ML2 show the most pronounced increases, although ML2 subsequently declines slightly. ML4 and ML5 demonstrate intermediate growth. In contrast, ML1 exhibits markedly slower growth throughout.

The calculated doubling times (Dts) further corroborate these observations. ML2 and ML3 present the lowest Dt values (21.50 h and 22.32 h, respectively), indicating the fastest growth rates among the tested lemon by-product concentrations. ML4 and ML5 follow closely (23.56 h and 22.56 h), while ML1 shows the highest Dt (32.59 h), consistent with its slower growth kinetics. These data suggest that ML2 and ML3 possess suitable lemon by-product concentrations, whereas ML1 is limited by slower substrate utilisation.

The production results (Table 6) showed a correlation between the substrate availability and microbial performance. The lowest concentration of lemon by-products (ML1) yielded limited biomass formation (5.204 g/L) and minimal PHBV production (0.134 g/L), likely due to carbon scarcity that restricted both growth and PHBV biosynthesis. As the concentration of lemon by-products increased from ML2 to ML5, a marked improvement in the dry cell weight (DCW) was observed, reaching a maximum of 21.346 g/L in ML5. PHBV accumulation also increased with substrate availability, peaking at 2.127 g/L in ML2, followed by a slight reduction in ML3 and ML4. These results suggest that increasing carbon availability supports growth, and polymer accumulation may not always increase proportionally.

The yield coefficients reveal distinct patterns of substrate utilisation among the tested media. ML2 exhibited the highest PHBV yield per substrate (0.216 g PHBV/g substrate) and the highest biomass yield (1.625 g DCW/g substrate), indicating efficient substrate assimilation supporting both cell growth and polymer synthesis. In contrast, ML3 showed a markedly lower biomass yield (0.585 g DCW/g substrate) but the highest PHBV yield per biomass (0.128 g PHBV/g DCW), suggesting a metabolic shift towards polymer accumulation under growth-limiting conditions. ML4 displayed intermediate biomass and PHBV yields, reflecting a more balanced carbon distribution.

Considering these results, ML2, and ML4 were selected for further research. ML3 was also retained, as it corresponds to the intermediate lemon by-product concentration between these conditions. Although its current PHBV production is lower, this medium is of particular interest because it requires less residue than ML4 and may, after suitable optimisation, achieve production levels comparable to those of ML2 and ML4. Despite the satisfactory results obtained with ML5, this culture medium was discarded, as the high concentration of lemon by-products did not significantly improve PHBV production, but the increased carbon is used by the cells for higher biomass production.

#### 3.1.2. Second Screening: Impact of Inoculum and Lemon By-Product Concentration on Microbial Growth and PHBV Production

To further optimise the process, a second experimental set was performed to assess the effect of the inoculum volume (2.5%, 5%, 10% *v*/*v*) at different lemon by-product concentrations (11%, 14%, and 17.5% *v*/*v*).

**Figure 2:**
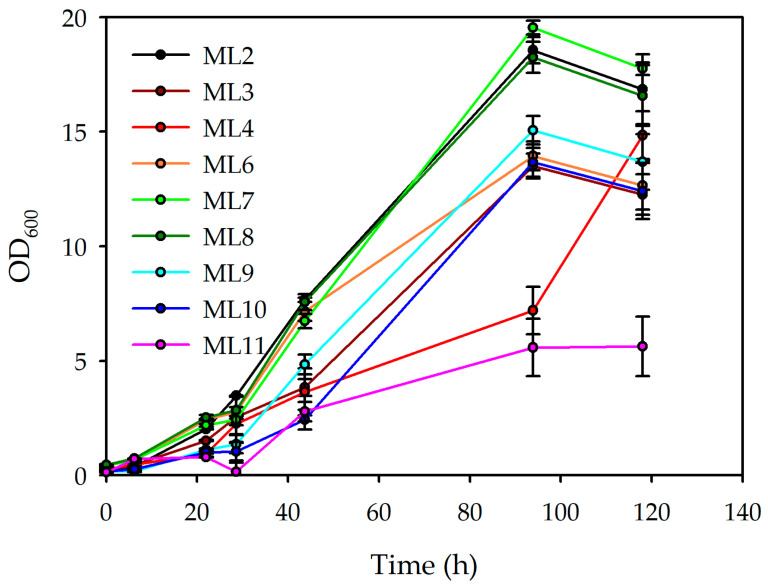
Growth curves and duplication rates obtained from different inoculum and lemon by-product concentrations.

The growth profiles (Figure 2) of the microbial conditions (ML2–ML11) show marked variability in both their growth kinetics and final biomass accumulation over the 120 h incubation period. During the early stages, all conditions follow a similar trend, characterised by a short lag phase and the onset of exponential growth. However, beyond this period, differentiation becomes apparent. ML7 and ML8 exhibit the most rapid and sustained increases, reaching their maximum values by approximately 100 h, indicative of superior growth performance. ML9 and ML6 also demonstrate robust growth. In contrast, ML3, ML10, and particularly ML11 display reduced growth, with ML11 achieving the lowest performance, suggesting slower adaptation or limited nutrient utilisation.

The doubling-time (Dt) values correspond closely with these growth trends. ML9 and ML10 exhibit the shortest Dts (20.25 h and 20.33 h, respectively), consistent with their faster exponential growth. These are followed closely by ML7 (24.14 h), ML3 (24.19 h), and ML2 (23.84 h), indicating similarly efficient cell division rates. ML4 and ML8 display moderately higher Dt values (25.68 h and 26.71 h), while ML6 (30.32 h) and ML11 (27.09 h) show the slowest doubling rates. Therefore, these findings suggest that ML9 and ML10 possess superior growth capacities, whereas ML6 and ML11 exhibit suboptimal performances.

These assays explored how varying the inoculum size at fixed concentrations of lemon by-products affected the biomass formation and PHBV production. Table 7 shows the results. The PHBV production results highlight the combined influence of the carbon availability and inoculum size on biomass formation and polymer accumulation. With a 5% inoculum (ML2–ML4), increasing lemon by-products from 11.0% to 17.5% enhanced the biomass (15.967 to 19.724 g/L), while PHBV production remained similar (1.883–2.127 g/L). The low PHBV yields (0.097–0.128 g/g) indicate that additional carbon predominantly supported growth rather than polymer synthesis. At a 10% inoculum (ML6–ML8), the overall performance improved. ML8 reached the highest biomass (22.603 g/L) and strong PHBV production (2.044 g/L), reflecting more effective substrate utilisation at higher cell densities. However, yields remained moderate, suggesting that excess carbon still favoured biomass over PHBV formation. Reducing the inoculum to 2.5% (ML9–ML11) altered the metabolic allocation. Although the biomass levels were lower, ML11 (17.5% lemon by-products) produced a comparatively high PHBV concentration (1.939 g/L) and the highest yield (0.278 g/g). This indicates that a low inoculum under carbon-rich conditions promotes stress-induced PHBV accumulation, likely driven by nutrient imbalance or redox pressure.

The yield coefficients obtained in the second experimental set further illustrate the strong influence of the medium composition on substrate utilisation and carbon partitioning. ML2 again showed the highest PHBV yield per substrate (0.216 g PHBV/g substrate), driven by its high biomass yield, confirming its efficiency in converting substrate into polymer. In contrast, ML11 exhibited the highest PHBV yield per biomass (0.274 g PHBV/g DCW) but the lowest biomass yield (0.304 g DCW/g substrate), indicating a pronounced metabolic shift towards polymer accumulation. ML7 and ML8 displayed more balanced profiles, combining relatively high PHBV yields per biomass with moderate-to-high biomass yields, resulting in competitive PHBV yields per substrate. ML6 and ML4 exhibited intermediate behaviour, with moderate substrate-to-biomass conversion and PHBV accumulation rates. Conversely, ML9 achieved the highest biomass yield (1.720 g DCW/g substrate) but one of the lowest PHBV yields per biomass, suggesting preferential substrate channelling towards cell growth rather than polymer synthesis. Overall, these results confirm that high PHBV accumulation per cell does not necessarily translate into the highest substrate-to-polymer efficiency.

Collectively, these findings indicate that using a 5% (*v*/*v*) inoculum with 11% (*v*/*v*) lemon by-products (ML2) represents the most effective condition for using lemon residues as carbon source to produce PHBV using a minimal culture medium. This condition is particularly advantageous, as a lower inoculum volume facilitates the scaleup process by reducing the volume required for seed cultures. Moreover, ML2 achieved the highest PHBV yield among the conditions evaluated.

#### 3.1.3. C:N:P Ratio Experiments: Effect of Nutrient Ratios (C:N and C:P) and Medium Composition on Microbial Growth and PHBV Production

This approach was designed to evaluate the combined effect of the culture medium type, nutrient molar ratios, specifically carbon to nitrogen (C:N) and carbon to phosphorus (C:P), and lemon by-product concentration on PHBV production. Two levels of lemon by-products, 11.0% and 14.0% (*v*/*v*), were assessed under both minimal and enriched conditions. The C:N and C:P ratios are described in Table 3 for each condition, and the inoculum size was maintained constant at 5% (*v*/*v*). This design allowed for an assessment of how the balance between carbon loading and limiting nutrients influences both biomass accumulation and PHBV biosynthesis.

**Figure 3:**
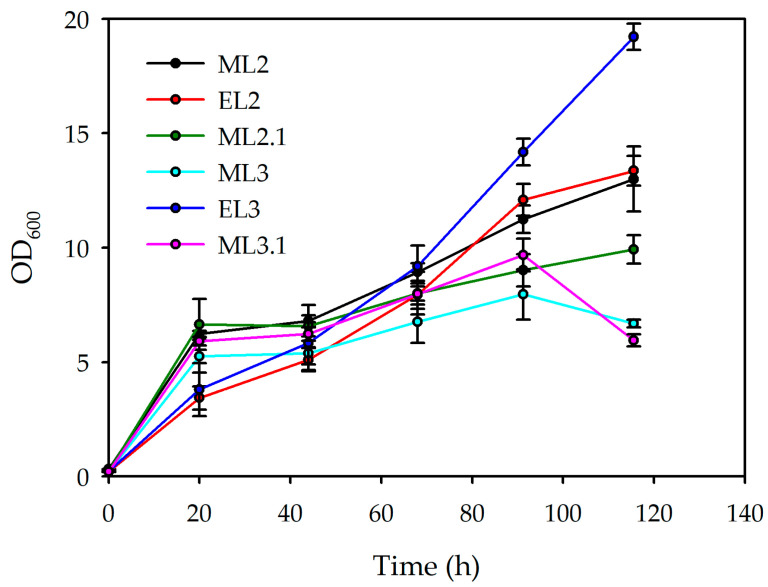
Growth curves and duplication rate obtained from each growth condition from the C:N:P ratio experimentation.

The growth dynamics (Figure 3) of the evaluated conditions (ML2, EL2, ML2.1, ML3, EL3, and ML3.1) reveal notable differences in both the rate and extent of biomass accumulation over the 120 h cultivation period. During the initial 24 h, all conditions display similar growth trajectories. However, from 40 h onwards, the curves begin to diverge. EL2 and EL3 exhibit the highest growth performances, indicating strong exponential growth and efficient nutrient utilisation. ML2 reaches an intermediate level, while ML3 and its derivative ML3.1 show more limited increases. ML2.1 demonstrates the weakest growth.

Dt data further support these patterns. EL3 (36.82 h) and EL2 (37.95 h) exhibit the shortest Dt values, corresponding to their rapid and sustained growth. ML2 shows a moderate Dt of 65.21 h, while ML3 and ML2.1 present considerably longer Dt values (83.81 h and 103.84 h, respectively). ML3.1 displays the slowest growth with a Dt of 114.24 h. These results collectively indicate that the enriched-medium conditions outperformed their minimal-medium counterparts, as a higher concentration of phosphorous and nitrogen improved the efficiency of cellular growth under the experimental conditions.

As Table 3 shows, a key comparison is established between the ML2.1 and ML2 conditions, both of which are formulated with 11% (*v*/*v*) lemon by-products and a C:N ratio of 7.17 but differ in their C:P ratios (200.03 and 40.01, respectively). In ML2.1, strong phosphorus limitation resulted in a DCW concentration of 8.312 g/L and PHBV production of 1.962 g/L. By contrast, ML2 showed higher biomass (15.967 g/L) and a slight increase in PHBV production (2.127 g/L). These results indicate that severe phosphorus limitation negatively affects both cellular growth and PHBV synthesis, thereby reducing overall productivity [42].

These observations are reinforced by EL2, which, despite the same 11% lemon by-products, provided a more balanced nutrient environment with a C:N ratio of 5.68 and a C:P ratio of 174.48. Under this condition, the DCW was lower than that in ML2 (7.072 g/L), but PHBV production increased to 2.564 g/L, as Table 8 shows. This demonstrates that enriched formulations can enhance PHBV biosynthesis by redirecting carbon flux towards polymer accumulation rather than biomass formation.

A similar trend was observed at 14% lemon by-products. Increasing the carbon load without adjusting the nutrient concentrations led to severe nutrient imbalance, resulting in markedly reduced biomass and PHBV production in ML3.1 (5.131 g/L DCW; 0.043 g/L PHBV) and ML3 (7.316 g/L DCW; 0.926 g/L PHBV). These results confirm that excess carbon alone does not enhance productivity.

By contrast, EL3, the enriched medium at 14%, achieved the highest PHBV production in this set (3.250 g/L) and the highest biomass concentration among the 14% conditions (9.080 g/L). This suggests that medium enrichment buffered the metabolic stress associated with high carbon loading, promoting efficient polymer synthesis while sustaining cell growth.

Enriched media (EL2 and EL3) achieved the highest PHBV yields per biomass and per substrate, confirming that these formulations favour polymer accumulation over cell growth. In contrast, minimal media such as ML2 promoted higher biomass formation but with lower intracellular PHBV content. ML3.1 exhibited poor performance in both growth and polymer production, underscoring the detrimental effects of nutrient imbalance.

Overall, these results confirm that maximising PHBV productivity requires a balanced nutrient stoichiometry, in which carbon availability is adequately matched with nitrogen and phosphorus. Enriched media such as EL2 and EL3 provide this balance, supporting both biomass formation and polymer accumulation, and therefore represent the most suitable conditions for PHBV production from lemon by-products.

### 3.2. Lemon By-Product Scaleup

For the scaleup fermentation, the EL3 culture medium was selected due to its superior performance, achieving a PHBV concentration of 3.250 g/L, the highest yield among the conditions tested.

**Figure 4:**
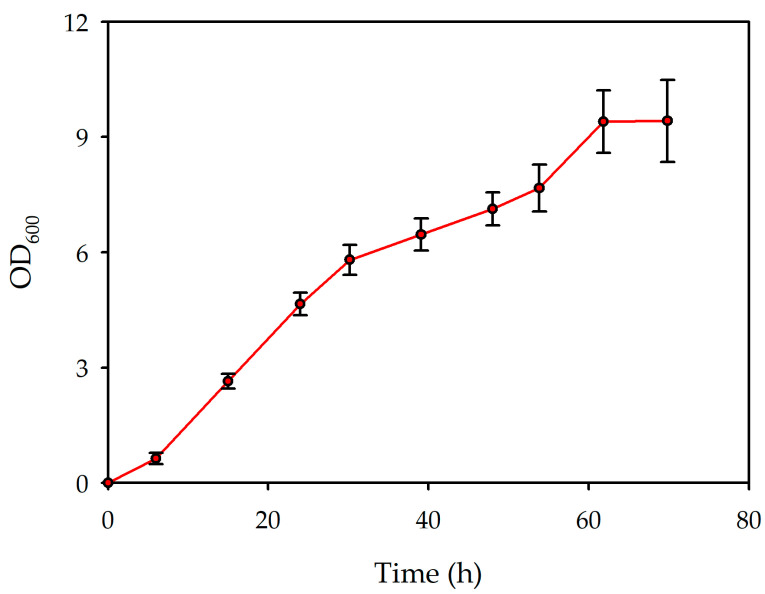
Growth curve obtained in scaleup cultures in the presence of lemon by-products.

The growth curve of the lemon by-product scaleup culture (Figure 4) exhibits a characteristic sigmoidal pattern, as in the other experiments. The initial lag phase is brief, followed by a pronounced exponential growth phase extending up to approximately 30 h. This phase indicates rapid cell growth and metabolic activity, suggesting efficient adaptation to the bioreactor.

Subsequent to the exponential phase, the growth rate gradually decreases, transitioning into a slower, linear phase between 30 and 60 h. By approximately 65–70 h, the curve reaches a stationary phase, indicating the cessation of net biomass increase.

The calculated duplication time of 25.26 h further supports a moderate growth dynamic under the conditions evaluated, consistent with the utilisation of complex substrates. These findings suggest that while the lemon by-product scaleup process supports substantial biomass accumulation, optimisation of the medium composition and aeration parameters could potentially enhance growth efficiency.

As Table 9 shows, the production of PHBV was assessed over a 72 h fermentation period. The DCW reached its peak at 48 h, measuring 10.388 g/L, which coincided with the highest concentration of PHBV recorded at 2.113 g/L, alongside a productivity of 0.030 g/Lh, biomass per substrate of 0.169 g/g, and PHBV accumulation per substrate of 0.169 g/g. By 72 h, although the DCW declined to 6.254 g/L, PHBV accumulation per biomass 0.297 g/g increased, indicating enhanced intracellular polymer accumulation. However, this increase in yield was accompanied by a reduction in PHBV productivity, which decreased to 0.026 g/Lh, likely attributable to diminished cell growth and metabolic activity.

In comparison, the 24 h trial demonstrated limited PHBV accumulation, yielding only 0.605 ± 0.033 g/L and the lowest yields, except in the gDCW/g substrate, suggesting that the process was still in its initial stages of biosynthesis. These findings indicate that a fermentation duration of 48 h emerges as the optimal timeframe for maximising the rate of PHBV production, while extended cultivation periods favour increased PHBV content relative to biomass. Consequently, the determination of the fermentation endpoint should be guided by the specific application, weighing the priorities of overall productivity against the efficiency of the polymer content.

The utilisation of lemon by-products for PHBV biosynthesis resulted in a polymer concentration of 2.113 g/L, demonstrating the potential of these by-products as a viable carbon source for PHBV production. This outcome highlights that waste derived from lemon processing can effectively support microbial growth and polymer accumulation, positioning it as a promising substrate within the context of valorising agro-industrial by-products.

After optimising the PHBV production process and the cultivation conditions to maximise the polymer yield, the next step is to evaluate the properties of the resulting biopolymer. This includes characterising the biopolymer after extraction, extruding it with nucleating agents (orotic acid, boron nitride, and theobromine), and comparing it with a commercial polymer such as Y1000.

### 3.3. PHBV Pure Characterisation

#### 3.3.1. NMR

**Figure 5:**
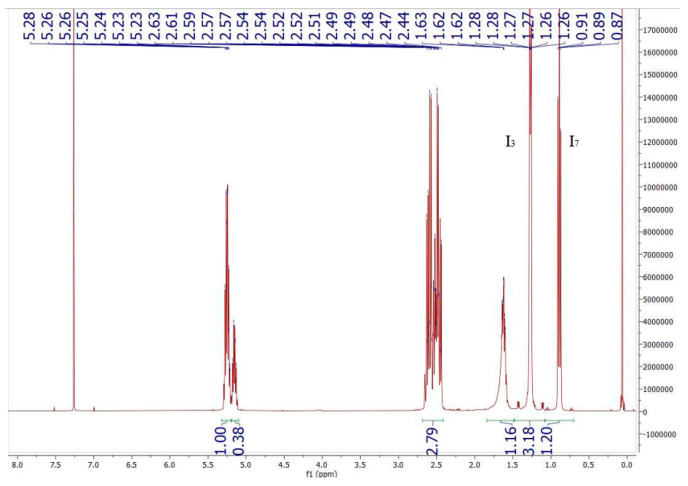
HFX PHBV NMR spectrum.

In Figure 5, the NMR spectrum of the polymer shows the values of the methyl proton integral attributed to the monomers 3HB (I_3_) and 3HV (I_7_). The 3HV percentage is calculated according to Equation (1): 3HV% = 1.20 × 100/(3.18 + 1.20) = 27.4%. According to its technical datasheet, the Y1000 polymer sample contains 1% 3HV, so an NMR was not necessary to determine the 3HV percentage.

#### 3.3.2. Thermogravimetric Analysis

**Figure 6:**
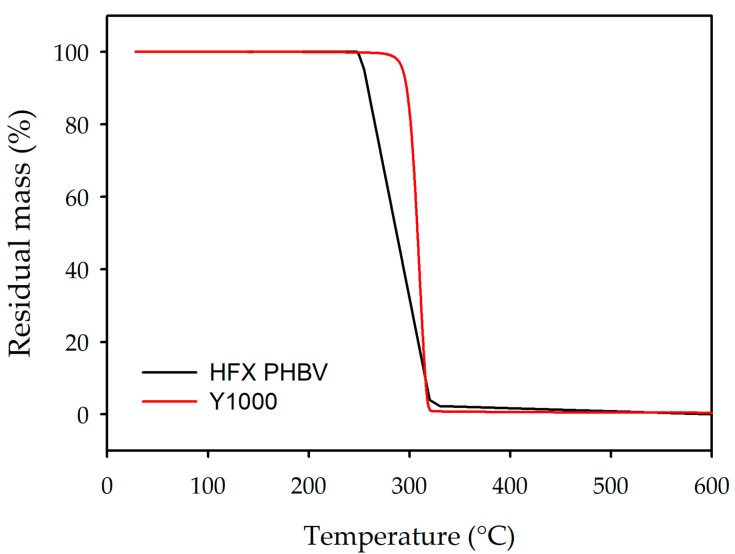
HFX PHBV and Y1000 TGA.

TGA reveals differences in the thermal stabilities between the homopolymer-like Y1000 and the high-3HV copolymer HFX PHBV. As shown in Figure 6, both polymers undergo a single-step degradation process, indicating a relatively homogeneous composition without multiple thermal phases [43] but also characteristic of random chain scission via cis-elimination reactions, a well-established mechanism for PHA thermal decomposition [44]. However, the thermal resistance profiles differ significantly due to their distinct macromolecular architectures. Y1000 exhibits superior thermal stability, with an onset degradation temperature (T_onset_) of approximately 300 °C, whereas HFX PHBV begins to degrade significantly earlier, at 260 °C. This 40 °C reduction in thermal stability for HFX PHBV is directly correlated with its high 3HV content (27.4%). Previous studies have demonstrated that bulky ethyl side groups from 3HV units acts as structural defects in the PHB lattice, thereby disrupting its crystalline [44]. The amorphous regions, being less dense and more accessible, require less energy to initiate the random ester cleavage associated with β-elimination, thereby lowering the T_onset_.

Furthermore, the degradation kinetics, evidenced by the slope of the mass loss curve, vary between the two grades. Y1000 displays a sharp, steep mass loss within a narrow temperature range. This behaviour is indicative of a highly crystalline material with a homogeneous structure, where the breakdown of the crystal lattice occurs cooperatively and rapidly once the activation energy is reached. In contrast, HFX PHBV exhibits a more gradual decomposition profile. This broader degradation window reflects its heterogeneous semi-crystalline nature, where the significant amorphous fraction degrades at slightly lower temperatures than the crystalline domains.

Finally, both samples show complete decomposition with negligible char residue (>350 °C), confirming the purity of the biopolymers. These results underscore that while HFX PHBV offers processing advantages (as seen in MFR), its lower thermal window requires tighter control during processing compared to the more thermally robust Y1000.

#### 3.3.3. Melting Flow Rate

The MFR is a fundamental rheological parameter employed to evaluate the flowability of polymer melts under standardised temperature and load conditions. Elevated MFR values are indicative of reduced melt viscosity, which typically correlates with enhanced processability during thermoplastic conversion. As HFX PHBV has a higher 3HV content, three different temperatures were tested and exhibited: an MFR of 0.12 ± 0.01 g/10 min at 140 °C, 0.69 ± 0.01 g/10 min at 145 °C, and 16.7 ± 0.5 g/10 min at 150 °C, whereas the Y1000 grade demonstrated a substantially lower MFR of 6.1 ± 0.6 g/10 min despite being measured at a higher temperature of 180 °C. Although tested at an elevated temperature, Y1000 exhibited a markedly lower melt flow rate, which is consistent with a comparatively higher melt viscosity and/or increased molecular weight relative to HFX PHBV.

Consequently, HFX PHBV can be regarded as exhibiting superior processability under the evaluated conditions, whereas Y1000 demonstrates pronounced resistance to melt flow, consistent with a higher melt viscosity and potentially greater structural integrity or molecular weight. This interpretation aligns with the extrusion profiles reported in Table 4, where HFX PHBV was processed at comparatively lower temperatures due to its enhanced melt fluidity from its high 3HV content. As previously discussed, PHBV is recognised as one of the most promising biopolymeric candidates for diverse industrial applications; however, its processing behaviour and mechanical performance impose significant technical challenges relative to conventional thermoplastics.

### 3.4. PHBV Blend Characterisation

#### 3.4.1. Differential Scanning Calorimetry

DSC was employed to evaluate the thermal behaviour of Y1000 and HFX PHBV, as well as their composites with different nucleating agents, focusing on the nucleation effect on PHBV crystallisation. Two heating cycles and one cooling cycle were performed to enable a meaningful comparison between neat PHBV HFX, neat Y1000, and both PHBV composites containing nucleating agents.

**Figure 7:**
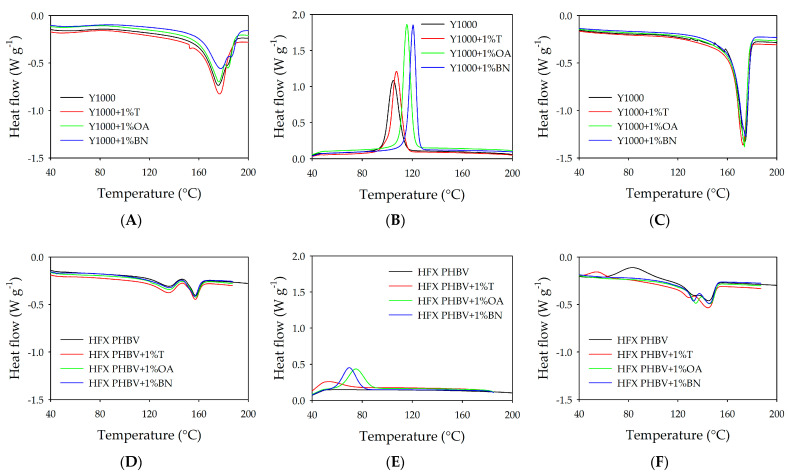
DSC thermograms of neat Y1000, HFX PHBV, and composites with nucleating agents. (**A**) Y1000 blends − first heating. (**B**) Y1000 blends − cooling. (**C**) Y1000 blends − second heating. (**D**) HFX PHBV blends − first heating. (**E**) HFX PHBV − cooling. (**F**) HFX PHBV − second heating.

As observed in Figure 7A–C, both the neat Y1000 and Y1000 + 1%T composites exhibited a single melting peak during the first heating cycle. This was likely attributable to the low proportion of 3HV comonomer, as indicated by the technical data provided by the manufacturer, and to the absence of a nucleating agent or the low effectiveness of theobromine. Thus, the low proportion of 3HV did not interfere with the lamellar structure of the 3HB comonomer matrix. On the contrary, orotic acid and boron nitride composites clearly show two melting peaks. In these cases, the effectiveness of the nucleating agent caused crystallisation of the 3HV comonomer, leading to structural changes at the edges of the 3HB comonomer’s lamellar structure [45]. This is generally interpreted in terms of melting–recrystallisation–melting behaviour during heating scans [46], which is detected by DSC. Another possible explanation is the one given by Miao [47], which says that the melting of two distinct kinds of crystal structures at different temperatures was the main cause of the multiple endothermic peaks. The thinner uniform lamellae with 3HV units’ complete exclusion melted at the lower melting peak, and the thicker sandwich lamellae with 3HV units’ partial inclusion melted at the higher melting peak.

However, the double melting peaks observed during the first heating cycle did not appear in the second cycle, neither for pure Y1000 nor for Y1000 compounds with nucleating agents. In this case, T_m2_ disappeared, while T_m1_ shifted to lower temperatures, and the enthalpy of fusion increased significantly for Y1000 + nucleating agents, suggesting that the better the nucleation effect of the nucleating agent, the higher the melting peak of fusion [48]. The T_c_ and enthalpy of crystallisation (ΔH_c_) were obtained from the DSC cooling runs and are summarised in Table 10 for composites containing different nucleating agents. For neat Y1000, T_c_ was approximately 107 °C; however, the incorporation of orotic acid and boron nitride significantly increased T_c_ to around 117 and 122 °C, respectively, whereas only minor changes were observed with theobromine. The upward shift of T_c_ indicated that the polymer crystallises more readily under lower supercooling conditions, suggesting a reduction in the energy barrier for crystallisation [47]. These findings also confirmed that the addition of nucleating agents substantially enhances the crystallisation rate of Y1000.

For HFX PHBV, with a higher 3HV content, two distinct melting peaks were observed in both the first and second heating cycles (represented in Figure 7D,F), with the peaks shifting toward lower temperatures during the second heating cycle. Moreover, these melting peaks were significantly lower than those recorded for the Y1000 polymer and its composites.

The presence of multiple endothermic peaks was attributed to the coexistence of two crystalline structures in PHBV: uniform lamellae, where 3HV units are completely excluded, and sandwich lamellae, where 3HV units are partially incorporated. As the temperature increased, the thinner uniform lamellae melted at the lower peak, while the higher peak was associated with thicker sandwich lamellae [49].

The overall decrease in the melting temperatures can be explained by the dependence of the melting point on the 3HV content [45]; as the 3HV fraction increases, the melting temperature is progressively depressed [50].

Regarding T_c_, no crystallisation peak was detected for HFX PHBV without a nucleating agent. However, T_c_ was clearly observed in the composites containing theobromine, orotic acid, and boron nitride (Figure 7E). A similar interpretation to that proposed for the Y1000 composites applies here: the presence of nucleating agents significantly enhances the crystallisation rate, with orotic acid exhibiting the greatest effectiveness in this case.

#### 3.4.2. Raman Spectroscopy

**Figure 8:**
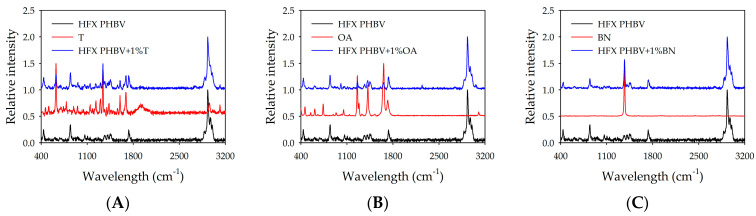
A comparison of the spot spectra obtained by Raman spectroscopy of the HFX PHBV blends. (**A**) HFX PHBV with 1% theobromine versus theobromine and pure PHBV. (**B**) HFX PHBV with 1% OA versus orotic acid and pure PHBV. (**C**) HFX PHBV with 1% boron nitride versus boron nitride and pure PHBV.

Raman spectra evaluate whether the additive remains present within the PHBV matrix, as well as its homogeneous dispersibility. Figure 8 shows the characteristic spectrum of PHBV, the additives, and the modified biopolymers by means of extrusion. Theobromine presented (Figure 8A) a characteristic peak at 626 cm^−1^, assigned to C–C deformation [51], C=C–N deformation [52], and δring(pyrimidine) (bending of the pyrimidine ring) + ν(N–CH3) (bending of said bond) + ρr (CH) (rocking of said bond) [53]. Orotic acid (Figure 8B) showed a peak at 1660 cm^−1^ due to ν(C=C) (stretching vibration of the C=C double bond in the ring) [54,55]. Finally, boron nitride (Figure 8C) presented a peak at 1372 cm^−1^, assigned to the E2g mode of h-BN (in-plane tensile vibrations of hexagonal boron nitride) [56,57]. The presence of nucleating agents in the polymer was confirmed, as characteristic peaks of the additives were present in the spectrum of modified biopolymers.

**Figure 9:**
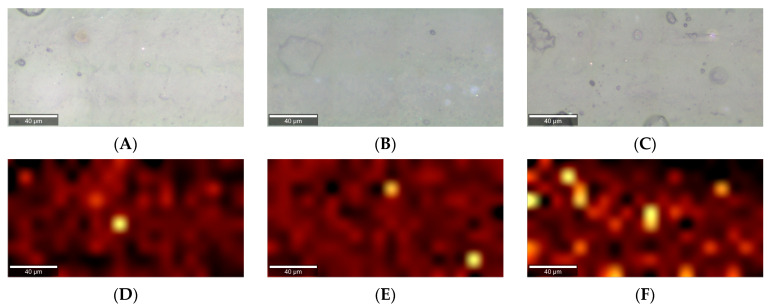
Raman pictures taken. (**A**) Mapping of the surface of PHBV + 1% T. (**B**) Mapping of the surface of PHBV + 1%OA. (**C**) Mapping of the surface of PHBV + 1% BN. (**D**) Superimposed spectral images of the surface of PHBV filaments with 1% T. (**E**) Superimposed spectral images of the surface of PHBV filaments with + 1% OA. (**F**) Superimposed spectral images of the surface of PHBV filaments with 1% BN.

Once the incorporation of the additives was corroborated, their dispersibility was inspected by performing a mapping on the extruded filaments in regions where surface heterogeneities could be observed. An averaged spectrum was taken at each position of a 20 × 10 matrix to identify the key features of each nucleating agent and examine their distribution on the surface. Figure 9 shows the superimposed spectra on the images. Chemical mapping was performed to generate images in which each pixel corresponds to the intensity of a selected Raman band that is characteristic of each additive. The maps were subsequently superimposed onto optical micrographs to establish a spatial correlation between the morphological features and chemical composition of the PHBV filaments. The results indicate that the additive was correctly added before extrusion, remained stable during the process, and did not agglomerate on the surface under any conditions.

#### 3.4.3. X-Ray Diffraction

Table 11 shows a decrease in crystallinity in HFX PHBV in comparison with Y1000, consistent with its higher 3HV content. Lower crystallinity in polymers is associated with lower stiffness and higher elasticity. OA, in contrast, appears to be the most effective nucleating agent, followed by boron nitride and theobromine. This behaviour is detected in both the Y1000 polymer and HFX PHBV.

**Figure 10:**
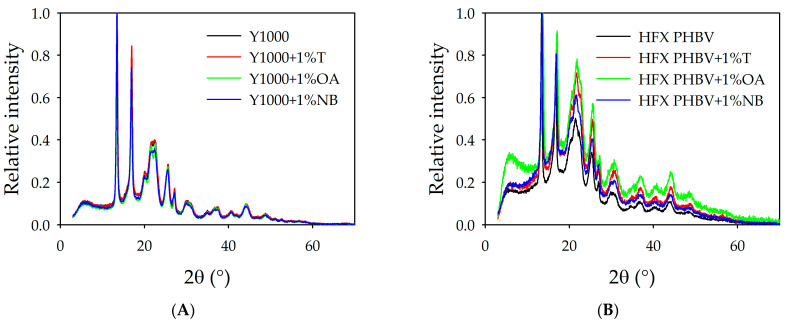
X-ray diffractogram WAXD patterns of blends. (**A**) Pure Y1000 and with different nucleating agents. (**B**) Pure HFX PHBV and with different nucleating agents.

In addition, a study of the unit cell size was conducted from the diffraction data, considering Bragg’s law and that PHB crystallises in the orthorhombic system. The different interplanar distances for the maximum diffraction angles, and from the interplanar distances, and the dimensions of the unit cell were calculated from Figure 10.

The calculated unit cells resulted in a smaller crystal size of Y1000 and PHBV with OA, as can be seen in Table 12. This reinforces the fact that OA is shown to be the most effective nucleating agent for both polymers.

Delving deeper into the nucleation mechanism of PHBVs, two classical nucleation mechanisms, namely, chemical nucleation and epitaxial nucleation, have been proposed and are widely accepted to elucidate polymer nucleation in the presence of nucleating agents [58,60,61,62,63]. For chemical nucleation, a chemical reaction occurs between the polymer and nucleating agent, leading to the formation of a new compound that can induce polymer nucleation [61]. In this specific case, the possibility of PHB reacting with the nucleating agents should be excluded.

The unit cell of PHB is reported to be orthorhombic with dimensions of a = 5.76 Å, b = 13.2 Å, and c = 5.96 Å [58]. In comparison, the triclinic unit cell of orotic acid has dimensions of a = 5.898 Å, b = 6.928 Å, and c = 9.592 Å [59].

The unit cell dimensions indicated a close lattice matching between PHBV (c = 5.938 Å) and orotic acid (a = 5.898 Å), with a difference of less than 1%. This structural alignment suggests that nucleation proceeds via an epitaxial mechanism instead of random crystallisation. Our hypothesis is that orotic crystals serve as effective templates for PHBV growth due to specific lattice coincidences [64], facilitating the ordered arrangement of polymer chains. The epitaxial relation leads to a decrease in the energy barrier for nucleation, promoting more uniform crystalline domains [63,65]. A similar explanation may also explain the nucleation behaviour of Y1000.

#### 3.4.4. Dynamic Mechanical Analysis

As shown in Table 13, the Young’s moduli exhibited a slight increase over time, suggesting secondary crystallisation and enhanced stiffness. After seven days, HFX PHBV blends did not reach 1000 MPa, indicating limited stiffness development despite the observed time-dependent behaviour. However, the incorporation of nucleating agents improved the stiffness, with HFX PHBV + 1% OA achieving the highest value (853 ± 51 MPa). Similar trends were observed for the other nucleating agents, with HFX PHBV + 1% T and HFX PHBV + 1% BN reaching 820 ± 56 MPa and 817 ± 20 MPa, respectively, confirming the effectiveness of nucleating agents in promoting crystallisation and increasing stiffness.

From a compositional perspective, these results can be directly related to the 3HV content of the polymer. Overall, the differences in the Young’s moduli confirmed that PHBV with its higher 3HV content is inherently less rigid than formulations with lower 3HV contents [66], which explains the comparatively lower stiffness of HFX PHBV and the beneficial role of nucleating agents in partially compensating for this effect.

## 4. Discussion

When compared with other reported substrates (Table 8), the PHBV concentration obtained from lemon by-products lies in the intermediate range. It exceeds the polymer yields derived from starch-rich confectionery waste (0.983 g/L [21]), ricotta cheese exhausted whey (0.868 g/L [67]), hydrolysed rapeseed meal (0.512 g/L [33]), olive mill wastewater (0.2 g/L [68]), and waste bread (0.526 g/L [69]). Furthermore, it closely matches the concentration achieved from silkworm excrement (2.1 g/L [70]), suggesting comparable nutrient availabilities and bioconversion efficiencies. However, higher PHBV production was achieved when using date waste extract (4.5 g/L [71]) and whey lactose (10.8 g/L [72]).

Table 14 shows that the control HFX PHBV sample did not exhibit crystallisation, in contrast to the formulations containing nucleating agents. This behaviour can be attributed to the high 3HV content of HFX PHBV, which disrupts chain regularity and hinders crystal formation, resulting in the absence of a detectable crystallisation peak. By contrast, the incorporation of nucleating agents significantly enhanced crystallisation, confirming their effectiveness at overcoming this structural limitation.

Among the additives evaluated, OA exhibited the strongest nucleating effect, increasing the crystallisation temperature to 75.50 °C and the crystallisation enthalpy to 26.53 J/g, followed by boron nitride and theobromine. These results support the proposed epitaxial nucleation mechanism of OA and highlight its superior efficiency compared with the other agents tested. When compared with the literature data, HFX PHBV and its nucleated variants showed higher crystallisation temperatures but lower enthalpies, indicating faster nucleation but a reduced overall crystalline fraction.

Overall, these findings demonstrate that the high 3HV content of HFX PHBV confers enhanced flexibility at the expense of crystallisation, while appropriate nucleation strategies enable partial recovery of the crystalline structure when required. This balance between flexibility and controlled crystallisation underscores the potential of HFX PHBV as a versatile biopolymer for applications requiring both thermal stability and mechanical compliance.

## 5. Conclusions

The production of PHBV from agrifood industry by-products using *Haloferax mediterranei* was revealed as highly effective, with optimised conditions (11% lemon by-products and 5% inoculum) yielding up to 2.127 g/L in minimal media and 3.250 g/L in enriched media (14% lemon by-products and 5% inoculum). Scaleup trials in a 2 L bioreactor confirmed robust productivity, peaking at 48 h with 2.113 g/L PHBV, while the nutrient balance (C:N:P ratios) was shown to critically influence yields. These results demonstrate that lemon by-products are a viable and sustainable carbon source for efficient PHBV biosynthesis, supporting their potential role in advancing circular bioeconomy strategies.

PHBV produced by *H. mediterranei* exhibited a favourable extrusion profile, as evidenced by a suitable melt flow index, allowing for efficient processing at lower temperatures and reducing the risk of thermal degradation compared with commercial PHBV with its lower 3HV content. Thermal and mechanical characterisation (TGA, DSC, and DMA) revealed suitable thermal stability and reduced stiffness, which was attributed to the lower crystallinity associated with the high 3HV fraction.

Furthermore, orotic acid, theobromine, and boron nitride were evaluated as nucleating agents in PHBV. Their nucleating efficiencies were compared between high-3HV PHBV and the commercial-grade Enmat Y1000. Both orotic acid and theobromine showed nucleating activity, but only orotic acid showed a performance comparable to that of the commercial boron nitride nucleating agent. The incorporation of nucleating agents effectively improved the crystallisation behaviour and mechanical stiffness, broadening the potential application range of the produced PHBV. This nucleating effect was also evidenced by increases in the non-isothermal crystallisation temperature and crystallisation enthalpy and changes in the unit cell parameters, as determined by XRD. Overall, orotic acid emerged as the most effective nucleating agent, providing the greatest enhancement relative to neat PHBV.

## Figures and Tables

**Table 1:** Lemon by-product composition provided by “Centro Tecnológico Nacional de la Conserva y Alimentación (CTNC)”.

Component	Concentration (g/L)
Fructose	26.67 ± 0.17
Glucose	43.60 ± 0.84
Maltose	9.20 ± 0.07
Sucrose	9.87 ± 0.70

**Table 2:** ML2-ML11 experiments with three different lemon by-product concentrations: 11, 14, and 17.5% (*v*/*v*) at three different inoculum concentrations: 2.5, 5, and 10% (*v*/*v*).

Condition	Lemon By-Products (*v*/*v*)	% Inoculum (*v*/*v*)
ML2	11.0	5.0
ML3	14.0	5.0
ML4	17.5	5.0
ML6	11.0	10.0
ML7	14.0	10.0
ML8	17.5	10.0
ML9	11.0	2.5
ML10	14.0	2.5
ML11	17.5	2.5

**Table 3:** Six experimental conditions for the exploration of a range of C:N (5.68–9.12) and C:P (40.01–254.59) ratios for PHBV production.

Condition	Culture Medium	Lemon By-Products (*v*/*v*)	C:N (g/g)	C:P (g/g)
ML2	Minimal	11.0	7.17	40.01
EL2	Enriched	11.0	5.68	174.48
ML2.1	Minimal	11.0	7.17	200.03
ML3	Minimal	14.0	9.12	50.92
EL3	Enriched	14.0	7.22	222.07
ML3.1	Minimal	14.0	9.12	254.59

**Table 4:** Temperature profiles used in extrusion.

Polymer	Zone 1 (°C)	Zone 2 (°C)	Zone 3 (°C)	Zone 4 (°C)	Die (°C)
Y1000	145	155	165	165	145
HFX PHBV	125	135	145	135	125

**Table 5:** DSC programs used to view crystallisation over time.

Polymer	First Heating	Isotherm	Cooling	Isotherm	Second Heating
Y1000	30–210 °C 10 °C/min	210 °C 1 min	210–30 °C10 °C/min	30 °C 1 min	30–210 °C 10 °C/min
HFX PHBV	30–190 °C 10 °C/min	190 °C 1 min	190–30 °C 10 °C/min	30 °C 1 min	30–190 °C 10 °C/min

**Table 6:** DCW (g/L) and PHBV (g/L) yields of the ML1-ML5 trials.

Trial	DCW (g/L)	PHBV (g/L)	Yield gPHBV/gDCW	Yield gDCW/gSubstrate	Yield gPHBV/gSubstrate
ML1	5.204 ± 0.354	0.134 ± 0.011	0.026 ± 0.003	1.239 ± 0.061	0.032 ± 0.01
ML2	15.967 ± 4.367	2.127 ± 0.025	0.098 ± 0.050	1.625 ± 0.080	0.216 ± 0.010
ML3	7.316 ± 1.302	0.926 ± 0.037	0.128 ± 0.004	0.585 ± 0.028	0.074 ± 0.003
ML4	19.724 ± 4.439	1.883 ± 0.107	0.097 ± 0.006	1.262 ± 0.062	0.120 ± 0.005
ML5	21.346 ± 1.615	1.152 ± 0.031	0.054 ± 0.013	1.195 ± 0.059	0.064 ± 0.002

**Table 7:** DCW (g/L) and PHBV (g/L) yields of the ML2-ML11 trials.

Condition	DCW (g/L)	PHBV (g/L)	Yield gPHBV/gDCW	Yield gDCW/gSubstrate	Yield gPHBV/gSubstrate
ML2	15.967 ± 4.367	2.127 ± 0.025	0.098 ± 0.050	1.625 ± 0.080	0.216 ± 0.010
ML3	7.316 ± 1.302	0.926 ± 0.037	0.128 ± 0.004	0.585 ± 0.028	0.074 ± 0.003
ML4	19.724 ± 4.439	1.883 ± 0.107	0.097 ± 0.006	1.262 ± 0.062	0.120 ± 0.005
ML6	8.855 ± 1.235	0.985 ± 0.273	0.122 ± 0.036	0.901 ± 0.044	0.100 ± 0.004
ML7	11.620 ± 0.117	1.782 ± 0.039	0.154 ± 0.039	0.929 ± 0.045	0.142 ± 0.006
ML8	22.603 ± 1.615	2.044 ± 0.003	0.122 ± 0.017	1.449 ± 0.071	0.131 ± 0.006
ML9	16.903 ± 0.163	1.404 ± 0.064	0.056 ± 0.006	1.720 ± 0.085	0.143 ± 0.006
ML10	17.938 ± 1.302	0.926 ± 0.037	0.128 ± 0.004	1.434 ± 0.071	0.074 ± 0.003
ML11	4.746 ± 0.208	1.939 ± 0.006	0.274 ± 0.001	0.304 ± 0.014	0.124 ± 0.005

**Table 8:** DCW (g/L) and PHBV (g/L) yields obtained from each growth condition from the C:N:P ratio experimentation.

Condition	DCW (g/L)	PHBV (g/L)	Yield gPHBV/gDCW	Yield gDCW/gSubstrate	Yield gPHBV/gSubstrate
ML2	15.967 ± 4.367	2.127 ± 0.025	0.098 ± 0.050	1.625 ± 0.080	0.216 ± 0.010
EL2	7.072 ± 0.940	2.564 ± 0.411	0.361 ± 0.011	0.720 ± 0.035	0.261 ± 0.012
ML2.1	8.313 ± 0.672	1.962 ± 0.071	0.237 ± 0.028	0.846 ± 0.041	0.200 ± 0.009
ML3	7.316 ± 1.302	0.926 ± 0.037	0.128 ± 0.004	0.585 ± 0.028	0.074 ± 0.003
EL3	9.080 ± 0.272	3.250 ± 0.014	0.358 ± 0.011	0.726 ± 0.035	0.260 ± 0.012
ML3.1	5.131 ± 0.223	0.043 ± 0.006	0.008 ± 0.002	0.410 ± 0.020	0.003 ± 0.001

**Table 9:** DCW (g/L), PHBV (g/L), Y gPHBV/gDCW, and PHBV productivity (g/LH) yields obtained in scaleup cultures in the presence of lemon by-products.

Condition	DCW (g/L)	PHBV (g/L)	Y gPHBV/gDCW	Y gDCW/ gSubstrate	Y gPHBV/gSubstrate	PHBV Productivity (mg/Lh)
24 h	7.542 ± 0.857	0.605 ± 0.033	0.080 ± 0.005	0.603 ± 0.029	0.048 ± 0.001	0.025 ± 0.001
48 h	10.388 ± 1.358	2.113 ± 0.030	0.205 ± 0.030	0.831 ± 0.041	0.169 ± 0.007	0.030 ± 0.001
72 h	6.254 ± 0.218	1.854 ± 0.011	0.297 ± 0.009	0.500 ± 0.024	0.148 ± 0.006	0.026 ± 0.001

**Table 10:** DSC thermal data of polymer blends.

	First Heating		Cooling		Second Heating	
Polymer Blend	AH_f_ (J/g)	T_m1_/T_m2_ (°C)	∆H_c_ (J/g)	T_c_ (°C)	∆H_f_ (J/g)	T_m1_/T_m2_ (°C)
Y1000	60.1	174.8	−65.6	106.7	67.6	172.1
Y1000 + 1%T	59.9	175.9	−70.4	109.9	76.8	171.4
Y1000 + 1%OA	60	175.4	−68.5	116.9	70	173.1
		182.8				
Y1000 + 1%BN	55.4	177.1	−72.5	121.8	77.7	171.9
		185.4				
HFX PHBV	16.9	135.2			24.6	133.1
		156.9				144
HFX PHBV + 1%T	20.8	134.8	−11	53.9	34.3	128.6
		156.6				143.6
HFX PHBV + 1%OA	20.3	135	−26.5	75.6	31	133.9
		157.1				145.1
HFX PHBV + 1%BN	22.8	135.3	−25.4	70.2	33.2	132
		155.9				144.6

**Table 11:** Comparison of crystallinities of two PHBVs by XRD.

Polymer Blend	Total Area	Crystalline Area	Crystallinity (%)
Y1000	936.06	658.98	70.4
Y1000 + 1% T	979.78	695.21	71.0
Y1000 + 1% OA	965.41	715.70	74.1
Y1000 + 1% BN	960.31	701.19	73.0
HFX PHBV	786.57	453.80	57.7
HFX PHBV + 1% T	650.77	362.16	55.7
HFX PHBV + 1% OA	355.53	219.85	61.8
HFX PHBV + 1% BN	715.1	375.75	52.5

**Table 12:** Comparison of unit cell dimensions of two PHBVs by XRD.

Material	a (Å)	b (Å)	c (Å)	Volume
Pure PHB [58]	5.76	13.2	5.96	453.15
Y1000	5.696	13.094	5.911	440.86
OA [59]	5.898	6.928	9.592	391.94
Y1000 + 1% T	5.680	13.038	5.957	441.15
Y1000 + 1% OA	5.696	13.086	5.911	440.59
Y1000 + 1% BN	5.712	13.131	5.921	444.20
HFX PHBV	5.748	13.183	5.938	449.96
HFX PHBV + 1% T	5.689	12.944	5.863	431.74
HFX PHBV + 1% OA	5.653	12.897	5.848	426.36
HFX PHBV + 1% BN	5.716	13.086	6.002	448.95

**Table 13:** DMA results using HFX PHBV with different nucleating agents.

Young’s Modulus (MPa)	Time (days)				
	0	1	2	3	7
HFX PHBV	383 ± 29	560 ± 26	610 ± 44	637 ± 38	687 ± 21
HFX PHBV + 1% T	480 ± 36	617 ± 70	710 ± 10	733 ± 32	820 ± 56
HFX PHBV + 1% OA	497 ± 23	650 ± 42	653 ± 114	717 ± 81	853 ± 51
HFX PHBV + 1% BN	517 ± 15	593 ± 42	697 ± 47	737 ± 72	817 ± 70

**Table 14:** Pure HFX PHBV and with nucleating agents—DSC cooling stages in the literature.

T_c_ (°C)	ΔH_c_ (J/g)	Polymer	Reference
n.d.	n.d.	HFX PHBV	This study
60.63 ± 10.47	12.57 ± 3.71	HFX PHBV + 1% T	This study
75.50 ± 0.10	26.53 ± 0.06	HFX PHBV + 1% OA	This study
69.70 ± 0.78	24.97 ± 1.02	HFX PHBV + 1% BN	This study
55.4	41.4	P(98.66% HB-co-1.34% HV)	[73]
61.0	48.7	P(99.54% HB-co-0.46% HV)	[74]
61.4	53.5	P(99.85% HB-co-0.15% HV)	[74]
58.6	36.1	P(99.22% HB-co-0.78% HV)	[74]
53.2	38.8	P(39.41% HB-co-60.59% HV)	[74]
58.0	49.0	P(99.50% HB-co-0.5% HV)	[74]

## Data Availability

The original contributions presented in this study are included in the article. Further inquiries can be directed to the corresponding author.

## Abbreviations

The following abbreviations are used in this manuscript:

3HB	3-hydroxybutyrate
3HV	3-hydroxyvalerate
ATCC	American Type Culture Collection
BN	Boron nitride
C:N	Carbon–nitrogen
C:N:P	Carbon–nitrogen–phosphorus
C:P	Carbon–phosphorus
CA	Cyanuric acid
CaCl_2_	Calcium chloride
CDCl_3_	Deuterated chloroform
CTNC	Centro Tecnológico Nacional de la Conserva y Alimentación
DCW	Dry cell weight
DMA	Dynamic mechanical analysis
DSC	Differential scanning calorimetry
Dt	Doubling time
FeCl_3_	Iron (III) chloride
*H. mediterranei*	*Haloferax mediterranei*
H_2_O	Dihydrogen monoxide
HFX PHBV	*Haloferax mediterranei* Poly(3-hydroxybutyrate-co-3-hydroxyvalerate)
HFX PHBV + 1% T	*Haloferax mediterranei* Poly(3-hydroxybutyrate-co-3-hydroxyvalerate) with one percent of theobromine as the nucleating agent
HFX PHBV + 1% OA	*Haloferax mediterranei* Poly(3-hydroxybutyrate-co-3-hydroxyvalerate) with one percent of orotic acid as the nucleating agent
HFX PHBV + 1% BN	*Haloferax mediterranei* Poly(3-hydroxybutyrate-co-3-hydroxyvalerate) with one percent of boron nitride as the nucleating agent
KCl	Potassium chloride
KH_2_PO_4_	Monopotassium phosphate
KNO_3_	Potassium nitrate
MFR	Melt flow rate
MgCl_2_	Magnesium chloride
MgSO_4_	Magnesium sulphate
NaBr	Sodium bromide
NaCl	Sodium chloride
NaH_2_PO_4_	Monosodium phosphate
NaHCO_3_	Sodium bicarbonate
NMR	Nuclear magnetic resonance
OA	Orotic acid
OD_600_	Optical density at 600 nm
PHA	Polyhydroxyalkanoate
PHB	Poly 3-hydroxybutyrate
PHBHHx	Poly(3-hydroxybutyrate-co-3-hydroxyhexanoate)
PHBV	Poly(3-hydroxybutyrate-co-3-hydroxyvalerate)
T	Theobromine
T_c_	Peak temperature of crystallisation
TGA	Thermal gravimetric analysis
T_m_	Peak temperature of melting
g/g	Gram per gram
*v*/*v*	Volume per volume
*w*/*v*	Weight per volume
Y1000 + 1% T	Y1000 polymer with one percent of theobromine as the nucleating agent
Y1000 + 1% OA	Y1000 polymer with one percent of orotic acid as the nucleating agent
Y1000 + 1% BN	Y1000 polymer with one percent of boron nitride as the nucleating agent
XRD	X-ray diffraction
ΔH_0_	Enthalpy of melting of 100% crystalline material
ΔH_c_	Enthalpy of crystallisation
ΔH_f_	Enthaply of melting
